# An overview of the material science and knowledge of nanomedicine, bioscaffolds, and tissue engineering for tendon restoration

**DOI:** 10.3389/fbioe.2023.1199220

**Published:** 2023-06-14

**Authors:** Wenqing Liang, Chao Zhou, Yanfeng Meng, Lifeng Fu, Bin Zeng, Zunyong Liu, Wenyi Ming, Hengguo Long

**Affiliations:** ^1^ Department of Orthopedics, Zhoushan Hospital of Traditional Chinese Medicine Affiliated to Zhejiang Chinese Medical University, Zhoushan, Zhejiang, China; ^2^ Department of Orthopedics, Zhoushan Guanghua Hospital, Zhoushan, Zhejiang, China; ^3^ Department of Orthopedics, Affiliated Hospital of Shaoxing University, Shaoxing, Zhejiang, China; ^4^ Department of Orthopedics, Shaoxing City Keqiao District Hospital of Traditional Chinese Medicine, Shaoxing, Zhejiang, China

**Keywords:** tendon injuries, bioscaffolds, nanomedicine, biomaterials, tendon reengineering

## Abstract

Tendon wounds are a worldwide health issue affecting millions of people annually. Due to the characteristics of tendons, their natural restoration is a complicated and lengthy process. With the advancement of bioengineering, biomaterials, and cell biology, a new science, tissue engineering, has developed. In this field, numerous ways have been offered. As increasingly intricate and natural structures resembling tendons are produced, the results are encouraging. This study highlights the nature of the tendon and the standard cures that have thus far been utilized. Then, a comparison is made between the many tendon tissue engineering methodologies proposed to date, concentrating on the ingredients required to gain the structures that enable appropriate tendon renewal: cells, growth factors, scaffolds, and scaffold formation methods. The analysis of all these factors enables a global understanding of the impact of each component employed in tendon restoration, thereby shedding light on potential future approaches involving the creation of novel combinations of materials, cells, designs, and bioactive molecules for the restoration of a functional tendon.

## Introduction

Tendon injuries (TI) may be triggered by trauma, but most result from cumulative tendon wear and tear due to misuse or age (Ning et al., 2023). A TI might appear to occur quickly but is typically the consequence of numerous microscopic rips that have occurred gradually with time (Amendola et al., 2022). The restoration ability of tendons is restricted, and scar tissue development is prevalent, resulting in poor mechanical qualities (Amendola et al., 2022). Medically, Achilles tendon recovery typically takes 4–8 weeks; nevertheless, a complete return to sports activities is not suggested until 4–12 months have passed.

New techniques depending on growth agents and stem cell transplantation have been developed to address these issues (Makuku et al., 2022; Yuan et al., 2023). Various preclinical studies demonstrate the capacity of a number of development factors to enhance the regenerative reaction and reduce scar development (Makuku et al., 2022; Yuan et al., 2023). In addition to stem cell transplantation and growth factors, novel tendon injury investigations concentrate on the creation of nano-material scaffolds (Zhu et al., 2022).

Tendons and their accompanying extracellular matrix are structurally made of materials with nanostructures. In current times, there is growing attention on the development of new nanomaterial for the redevelopment of the tendon. Nanotechnology involves the positioning, manipulation, evaluation, and modeling of matter composed of four to four hundred atoms. The region lower than 100 nm is significant because the basic laws of physics alter, resulting in new physical qualities that enable scientists to create new resources with precise features, such as strength and size, that exceed conservative bounds. It has been claimed that nanomaterials can enhance tendon restoration and reduce the formation of fibrous adhesions and scar tissue.

Nanoparticles (NPs) are materials having overall dimensions that are typically on the nanoscale. Recently, these substances have emerged as key actors in modern medicine, with therapeutic uses ranging from contrast agents in imaging to medication and gene delivery carriers into tumors. NPs function as a link between ordinary materials and atomic structures. Biomedical engineering has been researched for years. The key features (magnetizability, size, functionality) to be used to produce innovative therapeutic techniques in many clinical fields (size, magnetizability, functionality) (oncology, infettivology, radiography, nerve and tissue redevelopment, etc.).

We highlight the nature of the tendon and the standard therapies that have so far been used. Then, a comparison is made between the many tendon tissue engineering methodologies that have been presented so far, concentrating on each of the ingredients required to acquire the structures that allow for appropriate tendon restoration: cells, growth factors, scaffolds, and scaffold formation techniques. The analysis of all these factors enables a global understanding of the impact of each component employed in tendon restoration, thereby shedding light on potential future approaches involving the creation of novel combinations of materials, cells, designs, and bioactive molecules for the rejuvenation of a functional tendon.

## Tendon structure and mechanical characteristics

Tendons are fibrous connective structures whose primary purpose is to link muscles to bones and convey force (Duscher and Shiffman, 2019). They act as energy warehouses and aid in maintaining carriage and joint mobility (Canata et al., 2017; Qi et al., 2020), which necessitates that tendons endure significant tensile and compressive stresses (Duscher and Shiffman, 2019). Its activities are connected with distinct mechanical and physicochemical properties, which distinguish this tissue from all others in the body.

**FIGURE 1:**
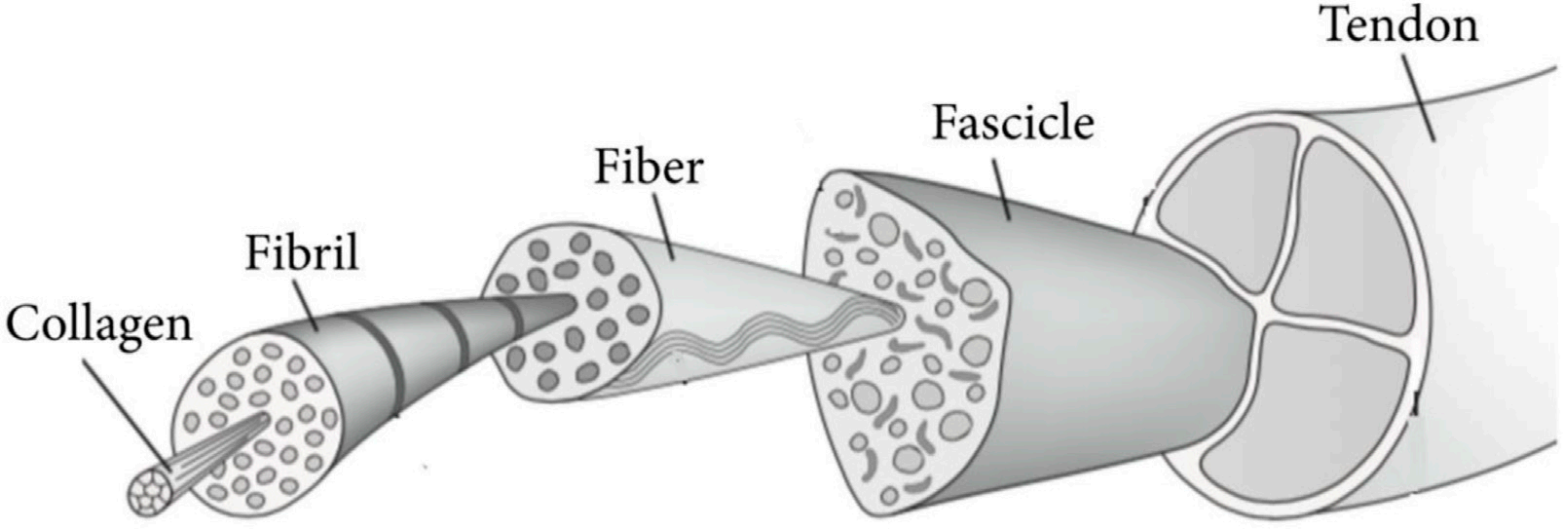
Structural of tendons. Adopted from Alshomer et al. (2018) under the Creative Commons Attribution License.

Tendons show a hierarchical structure at the macroscopic level (Figure 1) (Bosworth, 2011). As was previously said, they are continually expanding and constricting under varying tensile stresses. This form of movement is made possible by the orientation of the collagen fibers that comprise the tendons, their hierarchical architecture (subfiber, fiber, microfibril, and fascicle), the arrangement of their extracellular matrix (ECM), as well as the membranes or sheaths that protect the changed components. These latter ones permit fibers to move without generating friction (Duscher and Shiffman, 2019). Regarding the vascularisation of this tissue, there is a lot of diversity among the various tendon types. Tendons are always regarded as weakly vascularized tissue. The vasculature is mostly found on the tendon’s outside surface. Additionally, the blood flow is quite sluggish. As a result, and as subsequent research has demonstrated, this reduced blood flow leads to poor wound healing.

Tendons have limited cellularity and consist Tendons have few cells and are mostly made up of a water-rich ECM (55–70 percent) from a biochemical standpoint (Duscher and Shiffman, 2019). Aside from water, the matrix contains other substances for instance proteoglycans and glycosaminoglycans (GAGs) (1%–5% in terms of dry weight), elastin (1%–2% in terms of dry weight), and collagen fibrils (1%–2% of the dry weight) (60–85 percent in terms of dry weight). Collagen type I comprises about 80–90 percent of the total profile of collagen and is primarily accountable for the tendon’s characteristics. The fundamental unit of collagen I consist of two 1 chains and one 2 chain (Beck and Brodsky, 1998). Along with collagen I, other small collagen forms play fundamental roles in the formation and function of tendons. For instance, type II collagen (2 percent) and collagen type III (1–10 percent) are found in substantially lower levels in tendon tissues. In its place, elastin is accountable for a portion of the tendon’s unique elasticity. In the tendon, GAGs, glycoproteins, proteoglycans, as well as smaller molecules serve distinct roles. They reduce tissue distortion, contribute viscoelasticity, work as lubricants, and offer ECM integrity by occupying intrafibrillar space and avoiding collapse, among several other activities (Fang et al., 2023).

Tendons include multiple cell types with comparable properties, the most prevalent are tenocytes and tenoblast (tenocytes comprise 90–95 percent of tendon cells) (Duscher and Shiffman, 2019). Tenocytes are a kind of fibroblast cell characterized by their elongated form and stellate cross-section. Typically, they are sparsely distributed between the collagen fibrils in rows. They manufacture ECM elements and emit signals that govern tendon creation and growth (Sprague et al., 2022). Tendons also include an essential cell type known as tenoblasts. These are tendon cells in their immature state. Tenoblasts are extremely mobile and proliferative. Initially, the cells vary in shape and size; however, as people age, their morphology modifications, and they grow in length, more delicate, and more identical in shape, converting into tenocytes (Kannus, 2000; Benjamin et al., 2008). The other 5 to 10 percent of cells are a mixture of chondrocytes (set up in the bone junction zone), cells of the ancestor (tendon-derived stem cells, TDSC), vascular endothelial cells (surrounding the vascular system), lymphocytes, or other types of immune cells (for instance neutrophils, mast cells, and macrophages), smooth muscle cells and nerve cells (found close to muscle junction) (Canata et al., 2017).

**FIGURE 2:**
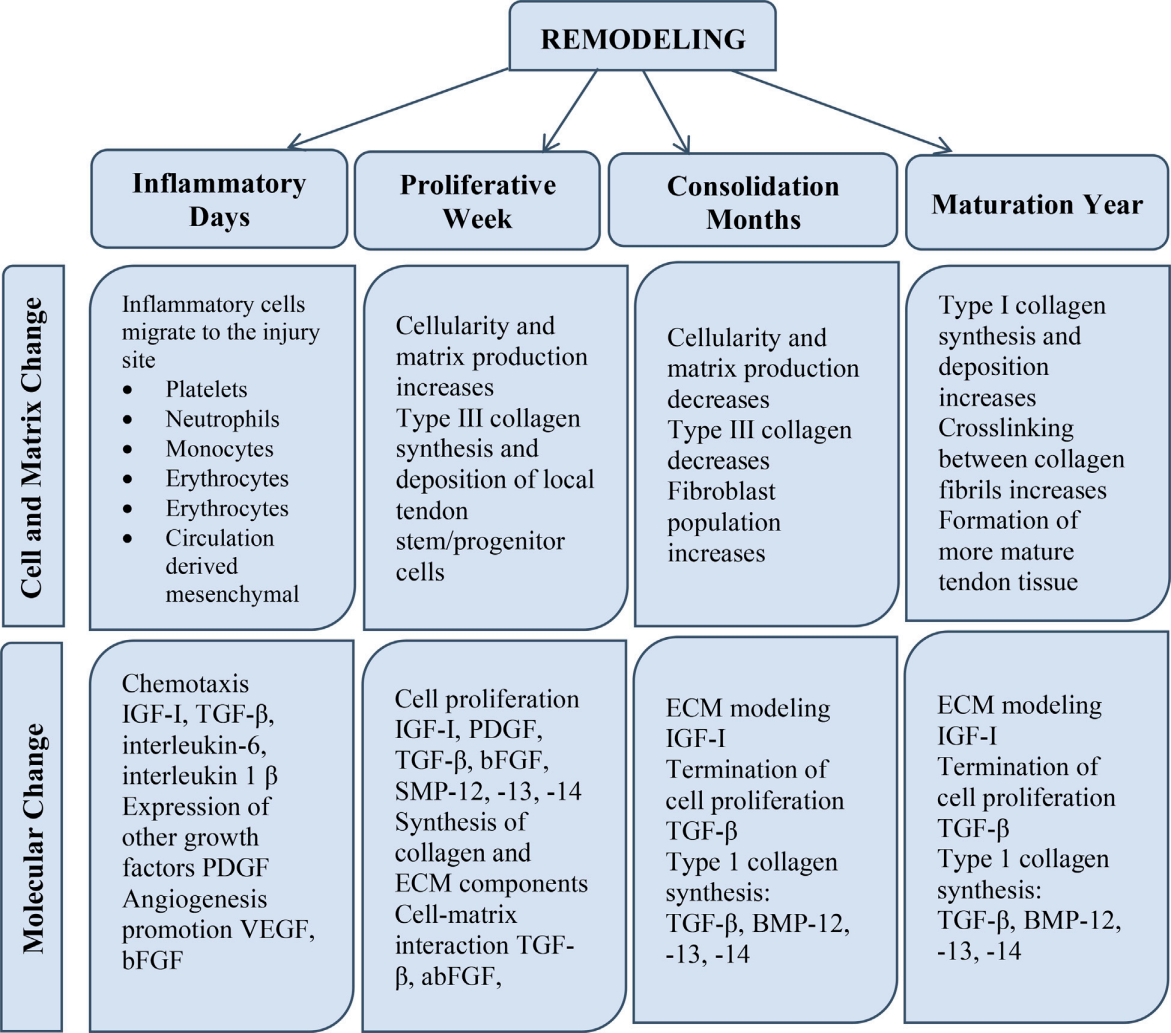
The phases of tendon restoration include the inflammatory phase, the propagative phase, and the remodeling phase. Alterations are classified according to their type as cellular, ECM, and molecular variations.

All of the aforementioned mechanical traits, including viscoelasticity, nonlinear elasticity, and anisotropy, are fully accountable for the unusual mechanical features of tendon tissues (Maganaris and Narici, 2005). The first mentioned characteristic of the tendon is its viscoelasticity. This characteristic enables it to regain its natural form when the stress that produced the distortion is eliminated. This behavior is possible because tendon tissues are highly resilient (Oliveira and Reis, 2017). The second attribute, nonlinear elasticity, relates to the stress-strain curve formed by putting different degrees of stress on tendons. Due to the tendon’s nonlinear properties (Figure 2), a strain-stress curve may be divided into three separate zones (Oliveira and Reis, 2017). The first is the area around the toes, which specifies tendon behavior for strain distortions up to 2 percent (low distortion). As the deformity worsens, the tendon transitions from the area of the toe to the zone of the linear (up to 4 percent of strain). Tendons in this area demonstrate elasticity and reversibility. If the displacement rate continues to rise, the tendon will approach its yield point (six percent strain) and the letdown area (8 percent of strain). In such circumstances, the physiological border of the tendon is exceeded (Wang et al., 2012). The distortion might reach a critical point, the point of collapse, at which even macroscopic breaks happen. The third attribute, anisotropy, states the variation in tensile strength that tendons can endure depending on the amount of force used (Maganaris and Narici, 2005; Choi et al., 2019).

## Tendon injuries and their different types

There are 02 primary forms of tendon injuries (TI), acute and chronic. Acute TI frequently develops in elderly individuals following repeated mechanical event failure and prolonged inflammation; a delayed diagnosis can lead to lifelong impairments (Gil-Melgosa et al., 2021; Tandogan et al., 2022). From time to time, acute TI, such as closed wrist TI, is managed with nonsurgical and traditional physiotherapy, whereas acute flexor tendon injuries are mainly treated with surgical assistance (Gil-Melgosa et al., 2021). It is widely established that recurring microtraumas in fibrotically-cured tendons are a frequent occurrence that might result in chronic stimulation and bursts (Tallon et al., 2001). Microtraumas are frequently linked to inflammatory conditions, which function fundamentally in tendon pathologies (Millar et al., 2017). Historically, inflammation has affected the categorization of tissue disease, however, the name ‘tendinosis’ has been acknowledged as oversimplification, and ‘tendinopathy’ is presently the most evocative phrase for the medical symptoms surrounding tendon problems (Abate et al., 2009). Furthermore, the healing development of tendons is also impacted by their structural placement and functions, and the normal repair procedures of spinner cuff tendon damage were reported to be often sluggish due to joint motion in multiple directions, hypo vascularization, and complicated anatomical framework (Dejardin et al., 2001). Figure 1 illustrates the Structural of tendons.

A normal tendon is a collagen network. ECM is thick, having a fibrillary network of mostly parallel-aligned type I collagen fibers. ECM includes tiny leucine-rich proteoglycans. In tendinopathy, tenocytes are thinner, and longer, have a higher nucleus-to-cytoplasm ratio, and generate less ECM but more type III collagen (mostly as a result of reduced deterioration). Histological analysis of the patient biopsy sample shows intratendinous collagen degradation, glycosaminoglycan, and fiber disorientation buildup between weakening fibrils and infiltrating inflammatory cells. Diseased tendons often have neovascularization and neoinnervation.

Achilles’ tendon is one of the tendon tissues most susceptible to injury due to the enormous forces that it is put through (Egger and Berkowitz, 2017). Trauma is the leading cause of Achilles TI, however, chronic injuries are also common. When the tendons are exposed to extraordinary trauma, acute Achilles’ tendon injuries are most common in young, highly active people, typically male athletes. Surgical therapy is suggested for individuals who wish to return to physical activity after recuperation, as the risk of re-rupture is lowest with the surgical intervention compared to conservative treatment (Romeo et al., 1999; Banala et al., 2018; Wang et al., 2023). Microtraumas and the absence of a normal healing response are regarded as the leading causes of Achilles tendinopathies. The process causing microinjury is unknown, but it is believed that it does not elicit a significant inflammatory response to expedite the typical normal healing process with three phases, hence causing tendinopathies ranging from mild to severe to whole burst (Dejardin et al., 2001).

Previous studies showed that on a cellular level, there is no variation in the response of tenocytes to mechanical load between cells isolated from various tendons, such as those linked with antagonistic muscles (Evans and Trail, 2001). Though, in a given tendon, the volume and duration of tensional stress induce distinct cellular responses in response to distinct stress patterns. Short periods of repetitive tension, for instance, stimulate cell proliferation, whereas longer periods inhibit it (Barkhausen et al., 2003).

The first report to identify MMP production in tendons described MMP-1 and TIMP-1 synthesis by human rotator cuff tendons in culture (Dalton et al., 1995). However, the study revealed no difference in the synthesis of enzymes or inhibitors between normal and deteriorated tissue. Gotoh et al. (1997) determined by immunohistochemistry that MMP-1 is localized at the margin of perforations in the supraspinatus tendon. Later, Riley demonstrated elevated levels of MMPs in the supraspinatus tendon relative to the distal biceps brachii tendon, which corresponded with increased collagen turnover in the supraspinatus tendon. Another study concluded that these alterations were the result of a repair or maintenance function in the more heavily laden supraspinatus tendon (Riley et al., 2002). Age is a possible risk factor for chronic tendinitis as it impairs tendon healing. The collagenase-injected SAMP6 group demonstrated increased expression of MMP-9, IL-6, and type III collagen, and decreased expression of TIMP-1, type I collagen, and TIMP-2, which are known to inhibit metalloproteinases (Ueda et al., 2019).

Tendinopathies restrict mobility and joint function, and they frequently cause disability and discomfort (Praemer et al., 1999). Tendon damage can occur as a result of a sudden injury (e.g., sports injury or laceration) or chronic impairment (e.g., degeneration or overuse damage), and the tendon’s ability to recover depends on the severity, duration, and location of the injury (Xu and Murrell, 2008). Tendon-to-bone integration is frequently necessary for the successful restoration of short and intracapsular tendons (e.g., rotator cuff tendons). Repairing lengthy and sheathed tendons effectively (e.g., flexor tendons) frequently depends on preventing repair-site gapping and maintaining tendon mobility (Dong et al., 2022). In general, tendon healing follows a typical wound-healing course. A brief inflammatory phase (on the order of days) is followed by a propagative phase (on the order of weeks), which is followed by a period of remodeling (on the order of months). Higher vascular permeability and an influx of local inflammatory cells such as macrophages, platelets, neutrophils, and monocytes, which emit chemotactic substances to attract blood vessels, fibroblasts, and intrinsic tenocytes characterize the inflammatory phase. In the propagative phase of the healing process, fibroblasts at the site of the wound proliferate and begin to produce collagen. Throughout the remodeling process, cellularity reduces and collagen becomes crosslinked and aligned with the direction of muscular force (Benjamin et al., 2008).

## Healing of tendon tissue through natural means

Because of the hypocellular and hypovascular characteristics of the tissue of the tendon, the triphasic normal therapeutic reaction is somewhat sluggish, thereby necessitating surgical intervention (Long et al., 2022). The three steps of the natural healing response are 1) inflammation, 2) propagation/restoration, and 3) remodeling (Li et al., 2023). Throughout the inflammatory process, the blood clot that forms soon after an injury serves as “preliminary scaffolding,” and burst tendon veins produce chemoattractants that attract migratory cells (neutrophils, monocytes, and lymphocytes) from the nearby tissues (Hafeez et al., 2021). Throughout this phase, phagocytosis breaks down necrotic debris, and tenocytes are activated/recruited. The second phase (the proliferative phase) occurs 2 days after an injury. Fibroblasts travel to the wounded region and begin proliferating at the epitenon, whereas intrinsic tenocytes from the endotenon and epitenon also migrate to the wounded area and begin proliferating. At this point, the number of neutrophils decreases while macrophages continually secrete growth factors (Maffulli et al., 2023). Tenocytes initiate ECM production with large levels of collagen type III, glycosaminoglycan, and water (Sharma and Maffulli, 2005; Sharma et al., 2011; Juneja et al., 2013). The damage occurs after 1–2 months, and the final remodeling stage begins. Augmented collagen type I is accompanied by reduced type III collagen, cellularity, and glycosaminoglycan at the wounded location. At 10 weeks, collagen fibers oriented towards anxiety/load and gradually transformed into tendon scar tissue, which certainly not achieves similar structural and mechanical characteristics as undamaged tissue even after 48 weeks (Hanff and Abrahamsson, 1996; Miyashita et al., 1997).

There are three key obstacles to natural healing: Sources of I cell infiltration, intrinsic (damaged tissue), and external factors (nearby tissues, for instance, synovial sheath). Particularly, external cellular infiltration aids in the creation of adhesions and scar-like tissue, which is structurally and biomechanically unusual and can result in a gap at the tendon-muscle interface (myotendinous), which has a significant impact on the muscle’s strength and motility (Gelberman et al., 1999). The resultant restored tissue exhibits aberrant thickness, form, and length, all of which diminish its functioning (Bruns et al., 2000), (Sharma et al., 2011). As a result of this, several studies concluded that operating interference is superior to conservative therapy since it significantly reduces nonfunctional scar formation (Walia and Huang, 2019), even though partial tendon rips may be recovered without operative surgery (Bruns et al., 2000; Huang et al., 2000; Kohler et al., 2013). Figure 2 shows the various mean of natural for the healing of tendons.

## Traditional treatment methods

**FIGURE 3:**
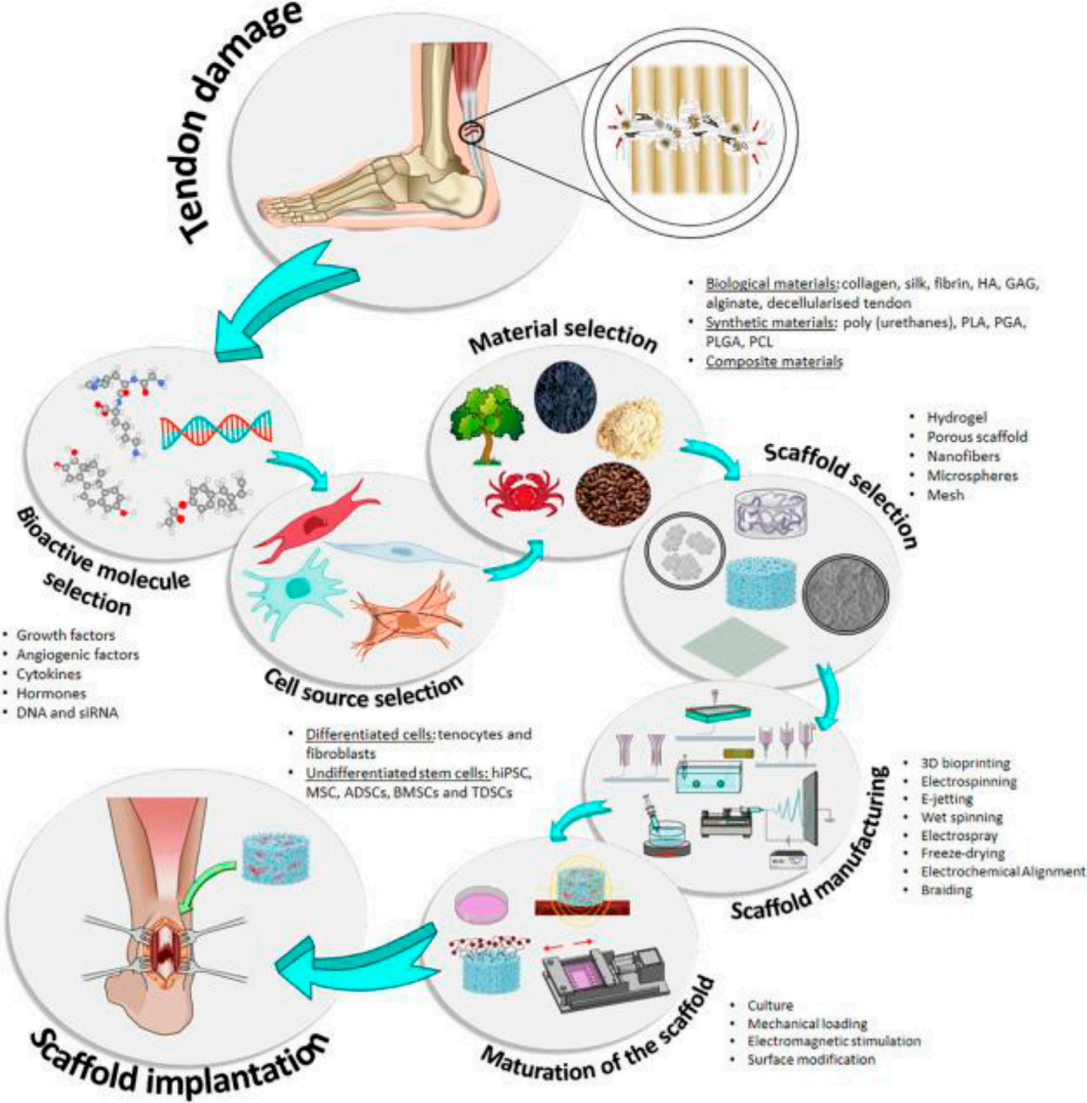
Diagram showing the primary components utilized in TI, including cells, scaffolds, and bioactive chemicals. Indicated are the stages of the conventional estimation for the use of TE. hiPSC, stimulated pluripotent stem cells; MSC, mesenchymal stem cells; ADSCs,r adipose-derived stem cells; BSCs, bone marrow stem cells; TDSCs , tendon-derived stem cells; HA, hyaluronic acid; GAG, glycosaminoglycans; PLA, polylactic acid; PGA, polyglycolic acid. Adopted from Ruiz-Alonso et al. (2021) under a Creative Commons license (Creative Commons CC-BY-NC-ND).

Presently, TI, both acute and chronic, is frequently cured also with traditional or operating interventions. Injections of corticosteroids, orthotics, rest, laser therapy, and ultrasound routinely utilize pain-relieving conservative therapies. Alternatively, surgical intervention may be indicated when conservative treatments fail to produce good outcomes (Dean et al., 2017; Wasker et al., 2023). In acute injuries, surgical treatments are common; however, when tried to compare to noninjured tissue, the reliability of the restored tendon is still lower in terms of function and structure, mostly due to unaligned collagen fibers and deformed ECM. In addition to damaged tissue and the inherent danger of operation, standard therapeutic procedures are linked with significant hazards such as adhesion development, nerve injury, infection, and the risk of other illnesses. In these endeavors, PT is frequently used with surgery to expedite healing and realign collagen (Fleming et al., 2005). In extreme situations, destroyed tissue is replaced using biological transplants. Autografts are a common method for repairing severely injured tendons, although they can induce functional impairment and significant morbidity at donor sites. Significant drawbacks of autograft treatment (Runer et al., 2023) include a mismatch in mechanics, necrosis, a lack of integration, as well as tissue laxity. Allograft is a substitute for autograft, but it has the same hazards of tissue rejection and infection transmission as autograft (Figure 3).

Since 1970, many FDA-approved commercial prosthetic devices have been offered as substitutes for autograft; nonetheless, continual contraction of muscle and mechanical stress limit prosthetic device uses as suitable alternatives (Chen et al., 2009). In reality, although the short-term results of these products are excellent, they are frequently accompanied by problems and uncertain long-term outcomes (Mascarenhas and MacDonald, 2008).

## Tissue engineering useful for tendon restoration

Tissue engineering (TE) blends biomaterials, biotechnology, and cell biology to restore or regrow body tissue (Liu et al., 2023). TE includes the selection or creation of resources utilized to construct scaffolds. These frameworks are coupled with cells (often stem cells) and physiologically active chemicals to produce structures (Figure 3) (Pearson et al., 2002) that help to rejuvenate, restore, or change human tissues or body parts. Because of scientific advancements in nanomaterials, cell separation, and culture, and the creation in addition to the isolation of development factors as well as other bioactive components, this new field has emerged and developed. All of these advancements have made it possible to develop biomimetic structures with features that closely resemble those of the original tissue (Reddy and Reddy, 2018; Bakhshayesh, 2019).

It is a very complicated field in which several factors must be considered, including the kind of tissue to be substituted or repaired, its position, structure, physical and chemical possessions, the existent types of cells, as well as their functions, the chemicals that comprise the extracellular matrix (ECM), etc. All these factors must be addressed when creating the scaffold, deciding its framework, components, the form or forms of cells to be comprised, the biologically active chemicals which may be required for the framework, and a variety of other factors (Caddeo et al., 2017; Eltom et al., 2019). The need to construct considerably more sophisticated structures whose functioning and biomechanical and structural properties and attitudes are much more related to biological structures that they substitute or repair is one of the primary problems facing tissue engineering (Peak, 2016; Armstrong and Stevens, 2020).

Thus, TE has established himself as a potential substitute for accelerating the redevelopment of injured tissues, such as tendons. TE for tendon restoration involves the fabrication of novel, healthy tissue to substitute or repair injured tendons (Lim et al., 2019). It is mainly focused on the formation of a suitable scaffold with physical and chemical and biomechanical characteristics as close to those of the original tendon as possible, ii) utilizing various cell types (fibroblasts, tenocytes, and distinguished cells) to mimic the cellular composition of the tendon, and iii) providing an atmosphere inside the scaffold that tries to promote tenocyte viability and ECM formulation (Hogan et al., 2011; Moshiri and Oryan, 2012).

## Materials used in novel tendon restoration techniques

### Resources for tissue engineering methodology

The goal of TE is to facilitate usual healing by developing *in vitro* synthetic grafts that may be placed into severely wounded areas (Youngstrom and Barrett, 2016). Synthetic grafts have a vital role in improving rehabilitation techniques and management of tendon restoration (Yin et al., 2016). Scaffolds have been the most frequently researched approach for tissue healing to date (Longo et al., 2012). Scaffolds and TE aim to avoid a relapse and speed up tendon recovery by reducing inflammation through mechanical support, making cell recruitment at the injured area, encouraging cell growth, as well as trying to stimulate ECM development and collagen fiber organization (Liu et al., 2008). Initial research supports the notion that scaffolds might offer a substitute for standard tendon augmentation procedures with great healing potential. However, insufficient data provide clear conclusions regarding the use of frameworks for tendon increase. In tendon tissue healing, the most desirable characteristics of scaffolds include cell attachment, proliferation, differentiation, ECM creation, metabolite transport, and collagen fiber alignment. The communication between seeded cells and scaffolding substances is crucial to functional scaffold design success. Preferably, scaffolding resources must drive regeneration procedures that provide a foundation for the optimal deposition of extracellular matrix (ECM) while simultaneously generating a reasonable rate of cell differentiation and proliferation (O’brien, 2011; Baldwin et al., 2018). There are now three primary types of scaffolds based on the materials used to treat serious tendon damage: Biological, synthetic, and scaffold composite.

### Synthetic scaffolding materials

Synthetic scaffolds consist of synthetic materials such as polyglycolic acid (PGA), carbon fibers, polybutyric polylactic acid (PLA), teflon, acid, and decaron, as well as biologically active glass. In comparison to scaffolds manufactured from natural materials (Cooper et al., 2005; Chen et al., 2009), they offer adequate mechanical qualities and a lower immunogenic response but restricted biocompatibility. Indeed, synthetic scaffolds are often more adaptable than biological scaffolds in place of physicochemical and structural features (Tsuji et al., 2003; Stoll et al., 2011) because they may be manufactured under precise circumstances. Though synthetic scaffolds give good potential outcomes, their absence of signaling molecules and mechanical fragility limit their extensive variety of tissue engineering applications (Liu et al., 2023; Rennekamp et al., 2023). Numerous polyesters, including PGA, PLGA, and PLA have been extensively studied for tendon healing. Glycolic acid and lactic acid are byproducts of their decomposition; they are bioactive components produced by the body that increase their biocompatibility. Cooper et al. (2005) revealed that PLGA is a suitable scaffold material for Achilles tendon healing. In addition, it was established recently that electrospun biomimetic PLGA tendon frameworks, which look like collagen fibers of the tendon extracellular matrix (ECM), could trigger an initial epithelial-mesenchymal transition (EMT) and tenogenic distinction of amniotic epithelial stem cells (AECs). The use of these stem cells made it possible to examine in detail the topological influence of the composites and the processes that allowed a cuboidal epithelial cell (often not articulating type I collagen) to develop into the mesenchymal tenogenic ancestry (Jin et al., 2018; Russo et al., 2020). These results revealed a favourable role for PLGA in tendon restoration, since PLGA exhibited adequate collagen synthesis and appropriate mechanical characteristics, elevated histology scores, and accelerated curative damage (Pelaz et al., 2017). PGA has also been suggested as a viable scaffolding substance for restoring the mechanical sturdiness of regenerated tendon tissue in a chicken model (Cao et al., 2018). The deprivation time of woven PGA scaffolds with superior mechanical performance has risen compared to unwoven PGA scaffolds (Riley, 2004; Rafiei and Haddadi, 2017). Regardless of belonging to the same category of polyhydroxyesters, the degradation profiles and cellular responses of PLGA, PLA, and PGA were highly distinct. Liu et al. (2011) and Liu et al. (2013a) discovered this difference by testing three distinct scaffolding resources: PGA, PLGA, and poly L-lactic acid (PLLA).

Poly—caprolactone is another synthetic substance utilized in tendon tissue engineering (PCL). These efforts created 3D hierarchical scaffolds cultured with human adipose stem cells (hASCs) and hTDCs using pure chitosan and PCL electrospun nanothreads (CANT). Using these aligned fiber scaffolds resulted in a tendon-like nano-to-macro architecture and increased tendon-related marker expression compared to the control for both types of cells studied (Laranjeira et al., 2017).

### Biological and composite scaffolding materials

**FIGURE 4:**
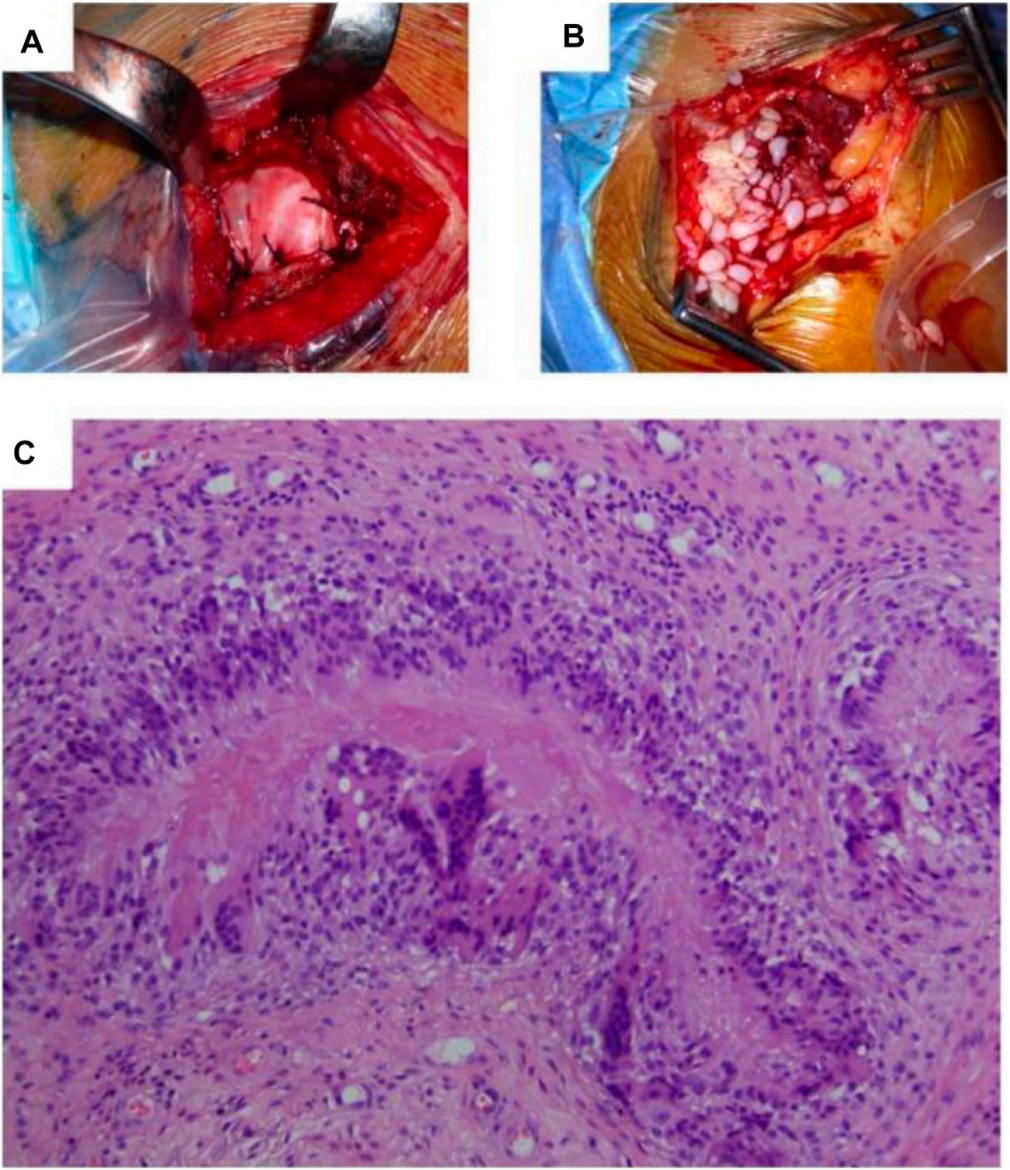
**(A)** Surgical scenario depicting the implantation of OrthADAPTTM during the original operation. **(B)** Surgical scenario depicting the removal of inflammatory tissue during revision surgery. **(C)** Histopathological examination indicated that granulomatous inflammatory alterations were the source of persistent inflammation resulting from an iatrogenic foreign-body reaction. The foreign substance is absorbed and encircled by immune cells structured as a palisade in the image’s center. Adopted from Lamas et al. (2019) under the terms of the Creative Commons Attribution 4.0 International License (http://creativecommons.org/licenses/by/4.0/).

The extracellular matrices of bovine, pig, equine, and human tissues are decellularized to provide biological scaffolds (Aamodt and Grainger, 2016; Kasravi et al., 2023). They were also derived from several biological substances, including fibrin, collagen, alginate, gelatin, agarose, hyaluronan, and chitosan (Zhang et al., 2023). Bio-BlanketW^®^, which is generated from the bovine dermis, OrthADAPT^®^, which comes from horse pericardium, and Restore^®^, which is manufactured from swine mucosa of the small intestine, scaffolds approved by the FDA that are now accessible for tissue tendon restoration (Chen et al., 2009). Figure 4 illustrates the implanted OrthADAPT™ during the operation. Dermis, pericardium, and intestinal mucosa are treated in these scaffolds by eliminating cellular and non-collagen elements (Chen et al., 2009; Chen et al., 2011). Scaffolds generated from the small intestine submucosa have been utilized effectively to treat Achilles tendon and spinner cuff damage (Little et al., 2010). Allografts can be recellularized *in vitro*, supplying suitable composites for tendon tissue healing (Tischer et al., 2007; Omae et al., 2009). These scaffolds offer various benefits over synthetic allografts, including strength, biomechanical stability, and natural structure (Tischer et al., 2007). Regarding cell proliferation, mechanical stimulation, attachment of cells, and metabolite transport, the extracellular matrix (ECM) of decellularized allografts resembles native tissue more closely (Little et al., 2010; Zhang et al., 2019).

Provided that collagen is the primary constituent of extracellular matrix (ECM), biological scaffolds generated from collagen are well-suited and regarded as a preferable alternative to polyester-based synthetic composite. These composites have been widely studied for tissue tendon renewal applications, displaying superior cell adhesion and propagation capacities related to synthetic scaffolds. Collagen gel has been described to improve the quality of healed patellar tendon injuries (Zhang et al., 2019). Though, collagen scaffolds have a lower mechanical sturdiness than polyester-based scaffolds. In an attempt to address this constraint, polyglyconate suture has been attached to collagen gel, resulting in enhanced biomechanical characteristics of the reconditioned patellar tendon in the hen model versus the control, albeit being significantly inferior to the undamaged tendon (Awad et al., 2003). In addition, physical assistance generated by mixing associated collagen fibers with a sponge or collagen gel revealed a greater capacity for the seeding of cells than arbitrary collagen gel (Juncosa-Melvin et al., 2006). Collagen sponges and fibers display greater mechanical strength when compared to collagen gel, as well as their use as scaffolding substances offers an additional attractive alternative to allografts and polyester-based scaffolds (Gentleman et al., 2006). In addition to the fact that collagen’s poor mechanical strength (Juncosa-Melvin et al., 2006) can be achieved through the use of other substances (Chen et al., 2009; Chen et al., 2014a), other limitations on the application of this polymer include its problematic characterization because of its many processability restrictions and its potential to elicit immunogenic responses (Lynn et al., 2004).

Chitosan, agarose, chitin, and alginate are also extensively explored for TE, despite their traditional use as scaffolding materials for hard tissue regeneration. They remained neglected in soft tissue engineering; nevertheless, they have lately attracted considerable interest as a potential scaffolding substance for the recovery of tendon and cartilage tissue (Funakoshi et al., 2005; Bagnaninchi et al., 2007). Mainly, chitosan has gained immense importance in soft TE as a scaffolding substance, particularly tendon redevelopment, due to its hydrophilic nature, outstanding mechanical power, and improved attachment of cell and propagation properties, especially in comparison to hydrophobic polyesters PLA and PGA (Suh and Matthew, 2000). Chitosan is a linear polysaccharide, chitin deacetylation, and comprised of randomly dispersed units of N-acetyl-D-glucosamine and -1–4-D-glucosamine. Chitosan is a viable option for use as a scaffolding material in tendon injuries due to its improved cell adhesion, differentiation, multiplication, structure that is extremely porous, and ECM synthesis. Particularly, chitosan was discovered to have improved biofunctionality due to the existence of N-acetylglucosamine, and the correspondence of glycosaminoglycan that offers growth factors and other proteins with increased adhesion potential (Suh and Matthew, 2000). Micro channeled highly permeable chitosan structures was constructed to manufacture patellar tissue of tendon, achieving optimum histological and biomechanical scores (Bagnaninchi et al., 2007). The mixture of chitosan with hyaluronan (HA), a key element of the ECM, boosted mechanical capabilities along with cell motility, differentiation, and adhesion (Funakoshi et al., 2005). The HA-chitosan scaffold improved the formation of type I collagen in spinner cuff tendon regeneration (Romeo et al., 1999; Funakoshi et al., 2005; Mouw et al., 2014).

Alginate can be used as a scaffolding material in conjunction with chitosan since it includes D-glucuronic acid, a glycosaminoglycan analogue with comparable biological activity. Chitosan-alginate hybrid scaffolds exhibited considerably improved cell attachment to tenocytes and ECM synthesis, mostly composed of type I collagen (Majima et al., 2005). Likewise, nanohydroxyapatite (n-HA) particles have been combined with chitin, fibrin, gelatin, PLGA, PLA, PCL, and polyamide-based composite tendon healing scaffolds (Moreau et al., 2005; Fan et al., 2009). These investigations have shown that integrating physiological and artificial biomaterials in hybrid composites is a potential technique for tendon healing (Chen et al., 2009; Chen et al., 2014a).

## Scaffold restrictions in tendon tissue regeneration

Even though scaffolds assure exciting results in the creation of tendon tissue, they have constraints that limit their use. Cell supply and *ex vivo* regeneration provide the greatest challenge for scaffolds (Goldenberg et al., 2021). Following cell seeding on a composite, they are restored in 02 ways: I in *ex vivo* reconstruction, bioreactor, and ii) via implanting within the body, *in vivo* restoration. In current years, attempts have been made to develop the tissue engineering industry by regenerating cells *ex vivo*. Indeed, manufacturing of mass-engineered tissues might provide goods that can be supplied to medical facilities on demand; but, the cells are not derived from patients, but rather from healthy, active people, which raises numerous safety concerns regarding the clinical applications of these devices. In addition, consistent harvesting, seeding, and maintenance techniques have not yet been developed, and *in vivo* and *in vitro* behavior of seeded cells differs (Alaribe et al., 2016; Bilodeau et al., 2020). To decrease the hazards of contamination and ailment transmission, it is necessary to standardize the safety evaluation of cell-seeded scaffold structures, even though human cells have never been marketed as a medicinal product. Moreover, a number of studies have documented limited diffusion of important *in vitro* incorporation of metabolites and products into scaffolds, drawing care to an additional significant issue that must be resolved: the *in vivo* neovascularization procedures (Cooper et al., 2005; Deb et al., 2018).

## Scaffold production with nanoparticles

Scaffolds, either synthetic or biological, have been developed to provide the tendon with mechanical support throughout the therapeutic procedure (Longo et al., 2012). Scaffolds are frequently utilized in conjunction with growth factors and stem cell regenerative medicine to provide structural (mechanical) as well as biological assistance for tissue repair. In TE, scaffolds may be functionalized via NPs to provide them with novel physical and chemical capabilities.

Karthikeyan et al. (2011) devised a technique for the production of functional biofibers composed of silk fibers (SF) covered with chitosan and permeated with AgNPs (Ag–C–SF). Chitosan [poly-b-(1–4)-D-glucosamine] is a recyclable, biocompatible, antibacterial, and sustainable polysaccharide with wide-ranging applications. Microbiological experiments, infrared spectroscopy, scanning electron microscopy (SEM), AFM examinations, and thermogravimetric analyses were used to examine the fibers. Antimicrobial action (NPs penetrate bacteria, limit ATP Synthesis, DNA denaturant, and disrupt the respiratory system) and enhanced thermal endurance were observed (Karthikeyan et al., 2011). The scientists note that this fiber may be a viable material for use in the healing of wounds and tendon restoration (Karthikeyan et al., 2011).

Liu et al. (2013a) investigated the behavior of AgNPs electrospun instantly into a degradable fibrous poly (L-lactide; PLLA) membrane. Due to their wide surface area and regulated porosity, electro-spun fibrous membranes are ideal blockades for the separation of tissue and drug administration to provide drug-loaded materials with a prolonged release period. The TEM micrographs of the fibers demonstrated that the AgNPs were effectively electrically turned into the PLLA fibers at varying concentrations and with the capacity to release Ag ions (Liu et al., 2013a). On fibroblasts, the anti-propagation impact of AgNP-loaded PLLA fibrous membranes was detected. Moreover, there was no cytotoxicity observed (Liu et al., 2013a). PLLA fibrous membranes loaded with AgNP exhibited broad-spectrum topical antibacterial efficacy against *S. aureus*, *P. aeruginosa*, and *S. epidermidis* (Liu et al., 2013a). These qualities make silver ions ideal for initiating anti-adhesion therapy and concurrently preventing infection. Moreover, even though cell propagation on the surfaces of PLLA fibrous membranes loaded with AgNP was lower than on PLLA fibrous membrane surfaces, the conventional negative impact of inhibiting cell proliferation was reclassified as a beneficial impact of inhibiting adhesion creation (Liu et al., 2013a). In reality, preventing bacterial adhesion must aid in reducing infections connected with medical devices. This *in vitro* investigation demonstrated that PLLA fibrous membranes loaded with AgNP inhibit adhesion of cells and growth without causing severe toxicity to cells (Liu et al., 2013a).

**FIGURE 5:**
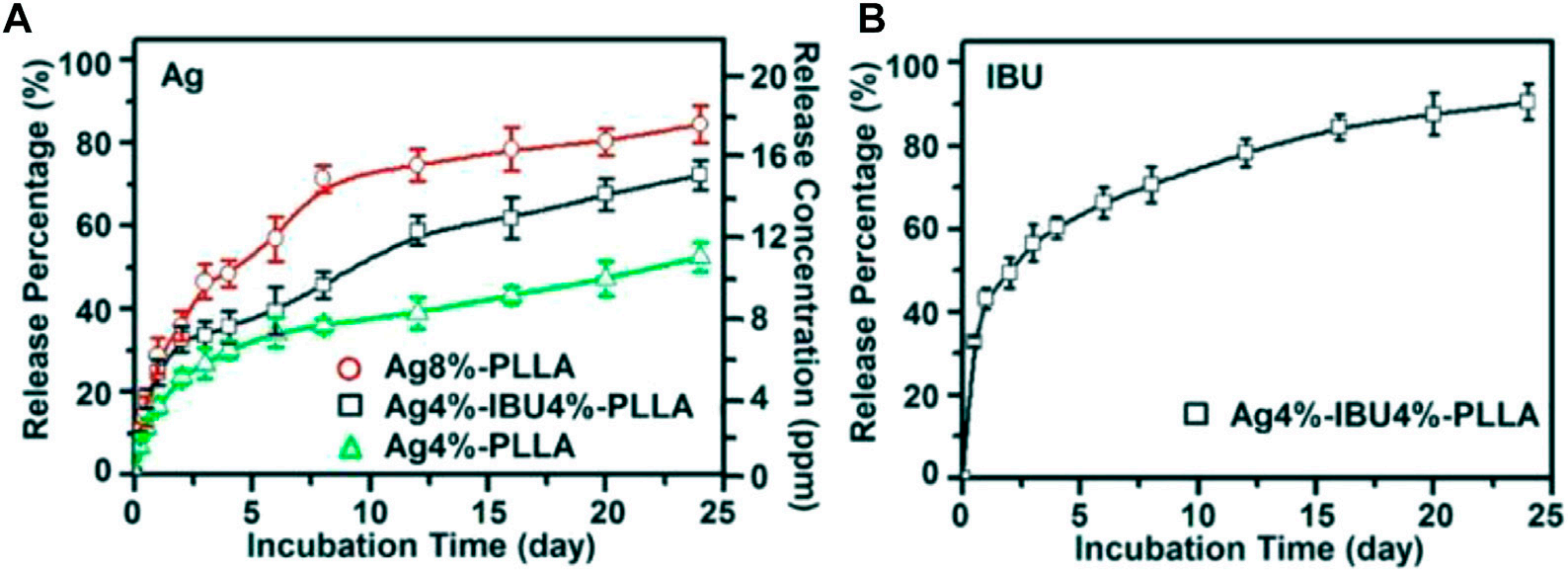
Ag ion cumulative release percentages and concentration from Ag 4 percent -PLLA, Ag4 percent–IBU4 percent–PLLA, and Ag8 percent–PLLA electrospun fibers **(A)**; and IBU release from Ag4 percent–IBU4 percent–PLLA electrospun fibers **(B)** following incubation in PBS at 37°C. Adopted from Chen et al. (2014b) under the terms and conditions of the Creative Commons Attribution license (http://creativecommons.org/licenses/by/3.0/).

Chen et al. (2014b) also utilized a fibrous membrane electrospun with AgNPs (Chen et al., 2014). They devised a mixture of Ibuprofen (IBU) and Ag to reduce the liver and kidney injury produced by a high dosage of Ag while preserving its anti-adhesion efficacy. Also established in this *in vitro* investigation was that not only the electro-spun Ag/IBU-loaded PLLA fibrous membrane inhibited adhesion and propagation of cells, but also decreased bacterial infection via the sustained IBU and silver ion release (Figure 5) (Chen et al., 2014b).

PLLA was densely packed with development factors in another work by Liu et al. (2013b) (heparin-binding site of basic fibroblast growth factor [bFGFs]; (Liu et al., 2013b). *In vivo* and *in vitro* investigations have revealed that bFGF stimulates angiogenesis, cellular differentiation, migration, propagation, and matrix formation in a number of tendons. Growth factors might be utilized to enhance tendon differentiation of cells, but their low biodisponibility *in vivo* is one of their significant limitations in clinical practice. Utilizing development factors in conjunction with scaffolds is one potential solution to this issue.

For tendon repair, a tubular scaffold of ZnO-loaded chitosan was created. After 4 and 8 weeks, macroscopically evaluating tendon adhesion and tissue reactivity to scaffolds. Histopathology also evaluates inflammation, angiogenesis, and collagen fiber organization. After 8 weeks, the scaffolds were entirely absorbed, tendon adhesions diminished, and histological investigations showed no significant tissue response or infection. Reduced adhesion development, enhanced gliding performance, and better histological features imply that this scaffold might cure acute tendon injuries (Yousefi et al., 2018). In a rabbit RC rip model, nanotopographic scaffold-augmented repair exhibited better healing than the control. Tendon ECM-like nanoscale structural signals of the tendon-inspired patch might trigger better aligned rejuvenation of underlying tissues, which involves the tendon-to-bone interface (Kim et al., 2020). Another study reported that scaffolds loaded with rPOSTN increase endogenous TSPC recruitment, tendon restoration, and renewel. Newly regenerated tendons restore mechanical characteristics and motility (Wang et al., 2021).

## Tendon healing nanoparticles (anti-adhesion effect, anti-microbial effect, and extracellular matrix arrangement modulation)

Silver nanoparticles have also been identified as antibacterial agents because they hinder ATP synthesis in microorganisms, deform DNA, and obstruct the respiratory chain (Klueh et al., 2000; Kumar et al., 2005; Morones et al., 2005). AgNPs are responsible for expediting burn wound therapeutic because of their antiflogistic properties, in addition to their antimicrobial properties (Klueh et al., 2000; Kumar et al., 2005; Morones et al., 2005).

Kwan et al. (2014) studied the possessions of AgNPs on tendon repair using Achilles Sprague Dawnley rats *in vivo* and *in vitro* (Kwan et al., 2014). The authors demonstrated *in vitro* that AgNPs stimulate the growth of important tenocytes to AgNPs and the synthesis of ECM elements (Kwan et al., 2014). In the *in vivo* testing, tensile testing demonstrated that the NPs treated group’s tensile modulus was much greater than that of the control group; however, it is substantially lower than that of a usual tendon (Kwan et al., 2014). The NPs of silver enhanced tendon repair and modulated ECM arrangement (more collagen fibrils of higher quality). This research demonstrated that AgNPs promote Achilles tendon repair by increasing cell propagation and encouraging the formation of proteoglycans and collagen (Kwan et al., 2014). The NPs of silver also exhibited an antiflogistic action, preventing the development of scar tissue and adhesions (Kwan et al., 2014).

**FIGURE 6:**
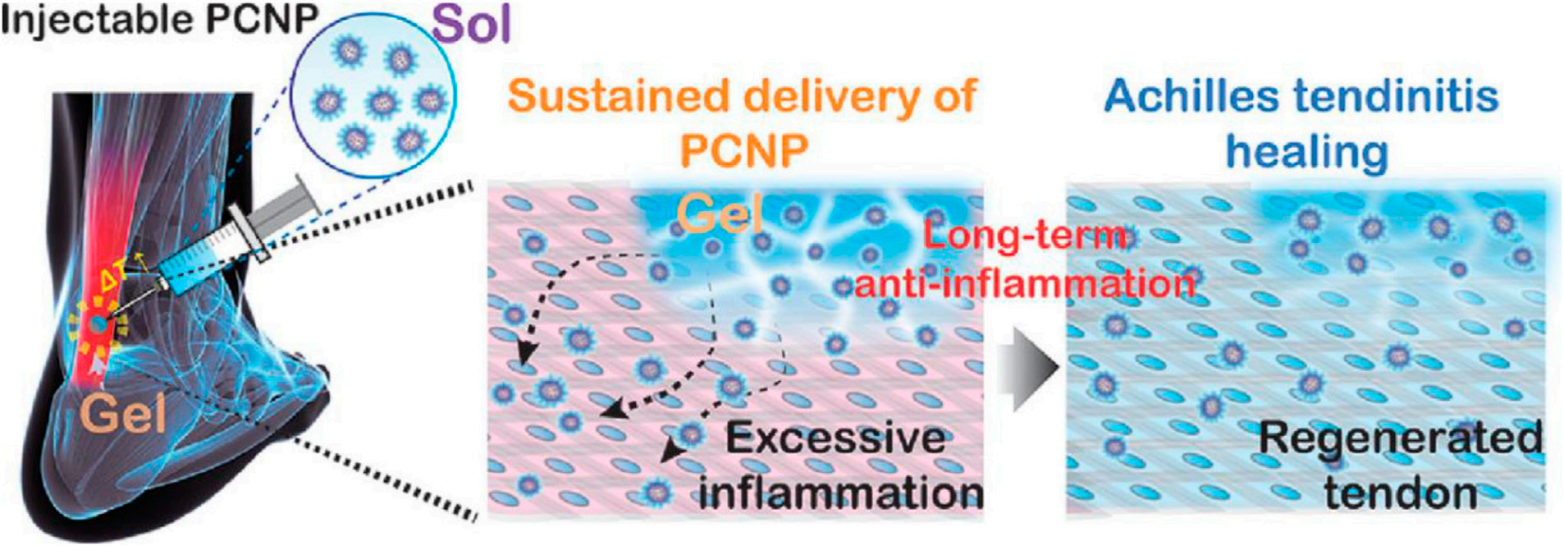
A schematic illustration of the injectable poly (organophosphazene)-celecoxib nanoparticle (PCNP) hydrogel for the non-invasive care for Achilles tendinitis. Adopted from Kim et al. (2022) under the terms and conditions of Creative Commons CC-BY-NC-ND license.

Empson et al. (2014) investigated the cellular and biomechanical response *in vitro* of nanoparticle-based treatment for injured connective tissues (Empson et al., 2014). They expected that the controlled and targeted biocompatible injection NPs into injured connective tissues could improve matrix mechanical characteristics, as indicated by an improvement in matrix rigidity as well as yield strength (Empson et al., 2014). The impact of NPs, specifically, single-walled CNCs and CNHs, on the mechanical characteristics of injured connective tissue were investigated. Empson et al. (2014) investigated the effects of NPs CNCs and CNHs on different kinds of connective tissues in their research, including porcine skin, which mimics the biological and mechanical characteristics of human tendons and ligaments, and porcine tendon as a model for treating target tissues. They examined data using dynamic light scattering (DLM), atomic force microscopy (ATM), cell cultures, and mechanical testing. This study’s findings demonstrate the potential of employing CNCs and CNHs to strengthen injured connective tissues nearby, the authors note (Empson et al., 2014). Due to the hydrogel’s biocompatibility and adequate pore size for the invasion of autologous cells, tissue rejuvenation was possible. Therefore, the celecoxib in the poly (organophosphazene) hydrogel was made to reduce chronic inflammation linked with the Achilles tendon through the long-term and sustained release of nanosized micelles, resulting in mechanically sufficient Achilles tendon restoration at the defect location (Figure 6) (Kim et al., 2022).

Current research has demonstrated that nanoparticles (NPs) can alter biological reactions (Cameron et al., 2022; Abbasi et al., 2023) and mechanical ECM characteristics (Cameron et al., 2022; Gao et al., 2023; Salem, 2023). NPs have been demonstrated to increase the mechanical characteristics of matrices of natural polymers, such as renewed cellulose, chitosan, and decellularized pig diaphragm tendon, along with inducing a cellular reaction (Qi et al., 2009; Deeken et al., 2011).

## MicroRNA delivery nanoparticles

The distribution of nucleic acid *in vivo* has been accomplished primarily through the use of viral vectors, despite certain patient safety issues (Vannucci et al., 2013). Numerous NPs have been produced using nanotechnology for use in therapy with genes to substitute viral vectors and prevent their negative effects (Raffa et al., 2011). Zhou et al. (2013) investigated the use of NPs as a non-viral vector for therapeutic purposes with genes to inhibit the establishment of peritendineus adhesion. The researchers stated that the miRNAs that inhibit the TGF-b1 expression (which causes fibrotic modifications and the creation of adhesions in tissues, including tendons) were implanted into plasmids, which were then loaded into PEI-polylactic-co-glycolic acid (PLGA) NPs to prevent peritendinous adhesion (Zhou et al., 2013). Intriguingly, scientists conducted their investigation both *in vitro* and *in vivo* using a similar tendon. The study of the data collected utilizing electron microscope imaging, molecular biology procedures, and biomechanical testing demonstrates the capacity of PLGA NPs as a new and effective supply of gene vehicles in tendons. In reality, miRNA transfection using PLGA NPs contributed to the suppression of TGF-b1 expressions, which stimulated tendon healing (Zhou et al., 2013). The outcomes of this investigation revealed that the cured tendons were weaker than those of the control group because of the suppression of TGF-b1 on cell migration, propagation, adhesion, apoptosis, and the creation of extracellular matrix (ECM) (Zhou et al., 2013). The scientists found that inhibiting the expression of TGF alone did not accomplish the required tendon therapeutic effect. It was hypothesized that trying to combine TGF-b1 miRNA plasmid with some other miRNA plasmids for other growth factor genes, to be supplied instantaneously by NPs, would provide superior tendon healing outcomes (Zhou et al., 2013).

Brevet et al. (2014) functionalized Mesoporous silica nanoparticles (MSN) with L-histidine and showed a greater transfection efficacy of histidine-functionalized MSN is more common than imidazole- or amino-functionalized MSN; the investigation was done *in vitro* and *in vivo* (Achilles tendon in mice; (Brevet et al., 2014). The outcomes demonstrated a high gene transfer effectiveness *in vitro*, while *in vivo* it was significantly lower (Brevet et al., 2014). Though, more research is being conducted to improve the potential of MSN as a nucleic acid delivery method to cure tendon wounds.

## Nanoparticles and tendon regeneration

There is a growing interest in synthesizing nanoparticles for tendon regeneration and therapy during the past decade. NPs are suggested as a promising discovery in tendon rejuvenation technologies in terms of gene therapy (as a gene shipper), drug delivery (growth factors), and propagation of cells, anti-adhesion, anti-inflammatory, and antimicrobial characteristic, as well as improved physicochemical and morphological properties of restored tissue (Parchi et al., 2016; Zhou et al., 2019).

Typically, NPs range in size between 20 and 600 nm. The NPs make it easier to connect with biological molecules inside the cell and on the cell surface in aspects that can be delegated to the physicochemical characteristics of cells (Mody et al., 2009). Their possible application in medication supply provides a number of benefits over current methods. To use NPs for medication distribution, these particles must be constant at the biocompatible, nanoscale, and guided to particular areas in the body following systemic injection. It might be accomplished by tying the particle to a ligand that binds specifically to the surface of the targeted cells. Moreover, NPs might bond selectively with therapeutic medicines, boosting the focus of healing molecules at the wounded tendon location (Sharma et al., 2006; Mody et al., 2009).

As nanometric delivery methods, nanoparticles can be employed to treat tendinopathy. For instance, they are possible to improve medication delivery via the skin using iontophoresis and phonophoresis. Both methods are generally utilized to cure inflammatory disorders associated with tendon wounds. In phonophoresis, high-frequency ultrasonic waves are utilized to provide medications, whereas, in iontophoresis, a low-voltage current is employed. Dohnert et al. (2012) and Dohnert et al. (2015) revealed that an enhanced supply of diclofenac diethylammonium using iontophoresis and phonophoresis with gold nanoparticles (AuNPs) as a drug transporter reduced the inflammatory reaction (decreased IL- and 1 TNF- levels) in an animal model of tendinopathy. The research concluded that AuNPs might increase the healing impact of iontophoresis and phonophoresis by enhancing drug distribution and anti-inflammatory synergy.

In addition, the NPs can be employed as non-viral nano-carriers for miRNA delivery *in vivo* in gene therapy and prevent the establishment of peritendinous adhesion (Arghiani and Shah, 2021). Zhou et al. (2013) demonstrated that PEI-PLGA NPs loaded with plasmid-implanted microRNA reduce TGF-1 expression. However, considerable repair of wounded tissue was not observed, indicating that simultaneous administration of TGF-1-miRNA and miRNAs of additional development factors is necessary. The suppression of TGF-1 led to cell propagation suppression, immigration, adhesion, and ECM secretion (Youngstrom and Barrett, 2016), resulting in weaker tendon strength than the control group. MSN containing L-histidine can also be utilized to treat tendinopathies. MSN improved the efficacy of histidine-functionalized NPs in cells transfected compared to amino-functionalized MSN or imidazole (Brevet et al., 2014).

Bioscaffolds can be combined with NPs to boost tendon healing tissue’s regeneration characteristics (Longo et al., 2012). P LLA fibrous membranes incorporated with dextran glassy NPs containing -FGF (dgNPs-FGF) were demonstrated to induce propagation of cells, segregation, angiogenesis, relocation, and ECM production *in vivo* and *in vitro* of tendons (Sprague-Dawley rat Achilles tendon) (Liu et al., 2013a). It was established that PLLA membrane loaded with dgNPs-FGF may controllably preserve the bioactivity of FGF to improve the quality of repaired tendon tissue.

## Conclusion and future perspective

Tendons are a type of tissue with unique qualities and traits that are intimately connected to their function in the body. Among these features, its viscosity and elasticity, poor cellularity, strong durability, and low vascularity stand out. In addition to other considerations, the extreme stress placed on these tissues annually causes millions of tendon harm globally. This is a big concern for healthcare systems worldwide and for people whose injuries severely impact their quality of life. The rehabilitation process for this sort of damage is extremely intricate, which is one of its major drawbacks. This is mostly attributable to the aforementioned tendon properties. In addition to being a difficult process, tendon regeneration occurs slowly and frequently resulting in nonfunctional or partially functional tissue.

Numerous therapies to regenerate the wounded tendons have been offered. Conservative therapies, operating therapies, managements with xenografts or allografts, nonsteroidal anti-inflammatory medications, healing depending on the infiltration of cells or growth factors, or therapy with genes stand out among others. Today, the most common therapy for severe cases of bursts is the combination of an early operational procedure with workouts for initial mobility. In a significant proportion of tendon wounds, it is impossible to restore the tendon’s pre-injury functioning or structure. In order to promote tendon redevelopment and restore its functioning, tissue engineering has been advocated for this kind of tissue.

Tissue engineering permits a more intricate strategy for tendon renewal. This field integrates molecular biology, substances engineering, and cell biology are being used to create structures that thoroughly resemble normal tissues. In tendon TE, numerous resources can be employed to create the scaffolds or structures that are intended. These substances can be categorized as either synthetic or biological. Everyone possesses their perks and downsides. The primary advantages of biological components are their bioactivity and biocompatibility, but their poor mechanical qualities are their primary drawback. In comparison, artificial substances often have excellent mechanical qualities but poor biological ones. Consequently, composite materials have grown in importance in recent years, as they pool the benefits of organic and artificial substances. As a result, several organizations utilize composite materials due to their superior biocompatibility, bioactivity, and mechanical qualities.

Another aspect utilized in tissue engineering is cells. There are two methods for tendon restoration: using both differentiated and undifferentiated cells. Tendon fibroblasts and tenocytes are the cells that have differentiated. Because most tendon cells are of this type, these cells are utilized to create scaffolds that are more similar to the genuine tissue. Their main drawback is that they are difficult to acquire, hard to nurture, and keep a low level of activity. There are more varieties of differentiated cells than pluripotent and embryonic stem cells, which are utilized infrequently because of ethical concerns and tenogenic risks. In contrast, ADSC and BMSC are the most frequently utilized stem cells because they are simple to collect, metabolically incredibly active, and straightforward to differentiate into tendon lineages. As was the case with chemicals, numerous sets have concluded that the ideal technique entails the combination of diverse cell lines.

Several biologically active substances with various roles are released into the extracellular matrix (ECM) throughout the tendon’s regular restoration. Additionally, these compounds can be incorporated into scaffolds to cure tendon damage. Growth factors have been the most researched and utilized of these variables. Its numerous roles include stimulating cell proliferation, improving the creation of various ECM elements, encouraging angiogenesis, and facilitating chemotaxis. The timing of their secretion and the role they play in tendon rejuvenation are becoming increasingly clear. Numerous research to date has included growth factors into scaffolds, often to promote the creation of type I collagen or the differentiation of cells into tenocytes. However, it should be emphasized that an increasing number of organizations are advocating using many growth factors concurrently. Monitoring and modulating the liberation of these development factors at the proper moment and combining all the development factors required for a complete repair of the injured tendon appears to be challenging.

The procedure utilized to get the scaffolds is also essential for the structure’s characteristics. The chosen material is intimately related to the technique used. Today, new processes have evolved, for example, electro-spinning, 3D printing, and electro-spraying. They enable the production of far more complicated scaffolds, improved regulators over the final structure, and automation of the structure creation procedure. This enables the production of frameworks with qualities more than in comparison to the original tissue. In addition, other research brings together these innovations to produce more complicated structures.

Analyzing the substances, cells, and growth regulators, and making procedures utilized in TE applicable to the restoration of tendons, it is clear that approaches involving the combination of these aspects yield more promising outcomes. The structures that most closely mimic the actual tissue and generate the highest renovation rates are the most complicated. In addition, the information that is now being developed in the area implies that it will be much simpler to adjust therapies and structures with each patient type and damage in the future.

Although significant progress has been achieved, the suggested approximations and results have enabled the formation of frameworks with features that are always too different compared to those of natural tendons. The configurations with the utmost performance to yet are primarily scaffolds or hydrogels having structural properties. The previous aid restoration in situations of minor TI is due to the influence of the growth factors and cells they comprise on the affected region. In the majority of situations, their sole role is to reattach the tendon’s ends that have been injured. While any of these 02 techniques may be near to clinical use, it is anticipated that a breakthrough in the creation of frameworks for tendon restoration will occur in the future years. This development must be geared toward the creation of structures that imitate the biomechanical characteristics of tendons. To solve the present paradigms, it is necessary to analyze and get more complex materials (via structural changes or appropriate material mixtures) and to build more complicated techniques using the current manufacturing processes. These manufacturing methods aim to mimic the hierarchical and fibrillar structure of tendons, either alone or in combination. Methods of utilizing manufacturing techniques, such as 3D bioprinting, to get fibers aligned in the same direction are already being investigated (the way in which the struggle will be conducted). This alignment of the fibers is a significant step in the direction of enhancing the mechanical characteristics of composites. Unquestionably, the repeatability of the structures generated and their sequential fabrication are crucial for the clinical use of these approaches. In this regard, contemporary manufacturing methods offer a significant improvement because they are extremely automatic.

Current therapeutic options are inadequate to return the tendon to its original form, making tendon damage a prevalent clinical concern. Tissue engineering solves this problem, with several techniques providing tendon repair with more mechanical strength than natural tendon regeneration.

Both artificial and natural scaffolds have been studied, and each has advantages and disadvantages. However, new research has generated mechanically robust scaffolds that have appropriate biofunctional qualities in animal experiments. The creation of nanocomposite scaffolds created from heterogeneous ingredients to more readily meet the demands of the regenerated tendon is a promising field for the future. Growth factors play a part in this regeneration process, but further study is necessary to explain their precise methods of contribution. In addition, a new study into growth factor combinations and gene therapy is anticipated to bear fruit in the future. In conclusion, mechanical stimulation is expected to be an intrinsic aspect of graft production for TTE, although additional shape and frequency tuning is necessary. With more refining and integration of these materials, tissue engineering demonstrates a very strong possibility for tendon repair.
